# Does normal fetal brain imaging in monochorionic twins following co-twin fetal demise eliminate the risk of adverse neurodevelopmental outcome?

**DOI:** 10.1007/s00404-025-08149-6

**Published:** 2025-08-22

**Authors:** Daphna Amitai Komem, Tally Pinchas Cohen, Shir Nov, Tal Weissbach, Irina Rabinovich, Yael Furman, Hagai Avnet, Boaz Weisz, Yoav Yinon

**Affiliations:** Fetal Medicine Unit, Department of Obstetrics and Gynecology, Sheba Medical Center, Ramat Gan, Gray Faculty of Medical and Health Sciences, Tel-Aviv University, Tel Aviv-Yafo, Israel

**Keywords:** IUFD, Fetal MRI, Neurosonogram, VABS, Monochorionic twins

## Abstract

**Objective:**

To assess the residual risk of long-term adverse neurodevelopmental outcomes in monochorionic twins following co-twin fetal demise and normal fetal brain imaging.

**Methods:**

All monochorionic twin pregnancies with co-twin fetal demise were included. Patients were identified through a search of inpatient medical records for co-twin fetal demise. Cases involving fetal demise following intrauterine procedures (e.g., laser ablation or termination of pregnancy following findings on fetal brain imaging) were excluded. Following co-twin demise, fetal brain imaging, including serial neurosonogram and fetal brain MRI, was performed on the surviving fetus. Neurodevelopmental outcomes were assessed postnatally using standardized age-appropriate developmental evaluation of personal–social, language, gross and fine motor skills, as well as hearing and vision screening. Additionally, parents completed a phone-based adaptive behavior questionnaire using the Vineland-II Adaptive Behavior Scales (VABS-II).

**Results:**

Nineteen patients met the inclusion criteria. Two patients underwent urgent cesarean delivery due to fetal distress following co-twin demise and did not undergo any imaging. Of the 17 patients who underwent fetal brain imaging, 2 had evidence of CNS injury, one of whom later presented with mild speech delay, and the other who achieved an adequate VABS-II score. Among the fetuses with normal brain imaging and available follow-up, 45.4% (5/11) had abnormal developmental evaluations including one global delay, one motor delay, one mild hypotonia, and two moderately low scores on VABS-II.

**Conclusion:**

Normal fetal CNS imaging following co-twin fetal demise in monochorionic twins does not guarantee normal neurodevelopmental outcome. This residual risk should be clearly discussed with parents during counseling to aid clinical decision- making.

**Supplementary Information:**

The online version contains supplementary material available at 10.1007/s00404-025-08149-6.

## What does this study add to the clinical work?

**Table:** 

Normal fetal brain imaging following co-twin demise in monochorionic twins does not eliminate the substantial residual risk of neurodevelopmental impairment of the surviving twin.	

## Introduction

Monochorionic twin pregnancies complicated by single fetal demise carry a substantial risk for long-term neurodevelopmental impairment in the surviving twin. The surviving co-twin faces a 26% chance of severe neurological injury, vastly higher than the 2% risk observed after a single-twin loss in dichorionic pregnancies [1–3]. This elevated risk stems from the unique placental anatomy of monochorionic twins—inter-twin vascular anastomoses allow acute transfusion of blood from the surviving fetus into the circulation of the demised twin at the moment of death, resulting in sudden hypotension and ischemia in the survivor that often leads to fetal brain injury.

A demise in the late second or third trimester tends to cause more profound hemodynamic imbalance, resulting in a higher risk for fetal brain injury [4]. Furthermore, uncontrolled spontaneous co-twin deaths confer a 37% risk of brain injury compared to a significantly lower risk following therapeutic interventions such as fetoscopic laser ablation, which reduce vascular anastomoses and mitigate acute transfusion insult [1, 5]. Underlying placental pathologies—namely twin–twin transfusion syndrome (TTTS) or selective fetal growth restriction (sIUGR) with unequal placental shares—also predispose survivors to an elevated risk of cerebral lesions. Extreme prematurity further increases the risk of adverse neurodevelopmental outcomes [5, 6].

Imaging remains the primary prognostic tool, though its ability to predict long-term outcomes is not well established. Prior studies have highlighted the superiority of brain MRI over neurosonography in detecting subtle and early cerebral lesions in monochorionic twins after fetal demise [7]. Early detection of cerebral ischemia within 72 h of fetal demise offers significant advantages by identifying early manifestations of cerebral ischemia that were undetectable on ultrasound [8]. These findings underscore the potential of MRI to provide critical early insights into the neurological risk of the surviving twin [7–11]. Despite these advancements, there remains insufficient information regarding the predictive value of fetal brain imaging for long-term neurodevelopmental outcomes in such cases. Therefore, the objective of this study was to evaluate the utility of fetal neurosonogram and brain MRI in predicting neurodevelopmental outcomes in monochorionic twins following fetal demise of one twin. Understanding the potential benefits of fetal brain imaging in this population may enhance clinical decision-making and improve prenatal counseling for these patients.

## Methods

This was a retrospective cohort study of all monochorionic twin pregnancies with co-twin fetal demise that were subsequently delivered in our institution. Patients were identified through a search of inpatient medical records for co-twin fetal demise. Exclusion criteria included fetal demise following any intrauterine procedure, i.e., laser ablation and termination of pregnancy following abnormal findings on fetal brain imaging.

### Management of IUFD in monochorionic twins

According to our center’s protocol, a measurement of the MCA‑PSV is performed following co-twin fetal death, and in case severe fetal anemia is detected in the surviving twin, intra-uterine transfusion is offered. We then offer diffusion-weighted imaging (DWI) within 72 h after fetal death to detect brain ischemic lesions^5^. Thereafter, ultrasound evaluation for the surviving twin including Doppler studies and brain neurosonogram is performed every 2 weeks until delivery, and fetal brain MRI is performed 3–4 weeks post-co-twins fetal demise to assess for brain hemorrhage or infarction [12]. The timing of delivery is individualized, aiming for 34–37 weeks of gestation. All neonates underwent head ultrasound during the early neonatal period.

### Data collection

The medical records of all subjects were reviewed and data regarding delivery outcomes were collected including gestational age at the time of delivery, mode of delivery, cord pH and Apgar scores, and short term neonatal outcomes.

### Developmental assessment

After delivery, surviving twins underwent developmental evaluations using two different tools. The first was a structured age-based developmental assessment routinely performed during well-child visits at the *Maternal Child Health Care Clinics* of the national health care system. This standardized protocol-driven assessment covered four developmental domains: gross motor, fine motor, language, and personal–social skills and as conducted in person by trained public health nurses. It provides objective information based on direct observation and has been validated in a large local pediatric cohort [13].

The second tool was the Vineland-II Adaptive Behavior Scales, Second Edition (VABS-II), which was completed by 13 families who consented to participate. VABS-II is a semi-structured interview administered to a caregiver and assesses adaptive behavior across domains such as communication, daily living skills, motor function, and socialization [14–16]. Lower scores indicate a higher level of impairment. Prior to contacting patients, a letter was sent informing patients that they will be contacted by phone with an option to refuse or consent. Developmental evaluations were performed by physicians, who were not aware of the fetal imaging findings. Due to variability in follow-up and participation, not all children completed both assessments. We report the findings of each tool separately to reflect these differences.

## Results

### Study population and delivery outcomes

During the years 2011–2021, 19 patients met the inclusion criteria. Their pregnancy and delivery outcomes are described in Table 1. Two pregnancies were MCMA. Of the 17 MCDA pregnancies, 8 were uncomplicated, 5 were complicated by TTTS, 3 by sIUGR, and 1 did not undergo monochorionic twin follow-up until the co-twin demise event. In the sIUGR cases, fetal demise occurred in the growth-restricted fetuses. In the TTTS cases, fetal death occurred in the donors. Mean gestational age at the time of fetal demise was 28.3 ± 4.2 weeks, and at birth 31.3 ± 3.5 weeks. Two cases had severe anemia and required intrauterine blood transfusion after the diagnosis of co-twin fetal demise.

**Table 1:** Pregnancy characteristics and delivery outcomes (*n* = 19)

MCMA twins	2 (10.5%)
MCDA twins	17 (89.4%)
Uncomplicated	10 (52.6%)
TTTS	5 (26.3%)
sIUGR	3 (15.7%)
Unknown	1 (5.2%)
Gestational age at the time of fetal demise	28.3 ± 4.2
Fetal anemia requiring IUT after co-twin demise	2 (10.5%)
Gestational age at birth	31.3 ± 3.5
Delivery < 28 weeks of gestation	6 (31.5%)
Delivery between 28 – 34 weeks of gestation	8 (42.1%)
Delivery > 34 weeks of gestation	5 (26.3%)
Mode of delivery	
Vaginal delivery	6 (31.5%)
Cesarean delivery	13 (68.4%)
Apgar 5 < 7	2 (10.5%)
Birth weight (median, IQR)	1012 (627.5- 1670)
Mechanical ventilation	3 (15.7%)
NICU admission	*N* = 9 (47.3%)

Data given as median (interquartile range) or mean ± SD

*MCMA* monochorionic, monoamniotic, *MCBA* Monochorionic, diamniotic; *TTTS* twin-to-twin transfusion syndrome, *sIUGR* selective intrauterine growth restriction, *IUT* intrauterine transfusion

Two newborns died after delivery; one was born at 25 weeks with IVH grade 4. The other was born at 26 weeks with severe RDS and died at the age of 10 days. Both had a normal fetal neurosonogram and no fetal brain MRI was performed.

### Fetal brain imaging

Seventeen underwent brain imaging after co-twin fetal demise: all patients underwent neurosonogram and 15 underwent fetal brain MRIs (10 DWI-MRI, 14 MRI 3 weeks post-demise). Two patients had abnormal findings on ultrasound and MRI. One patient demonstrated a unilateral acute infarction in the caudate nucleus and enlarged cisterna magna, shown on MRI 3 weeks after fetal demise. The second patient had lateral ventricles asymmetry measuring 7 and 10 mm with irregularity and a signal suggesting Grade I IVH, detected on DWI-MRI 72 h after fetal demise. Fifteen out of 17 (88.2%) patients had normal fetal brain imaging. During the early neonatal period, all patients underwent head ultrasound. Abnormal findings were observed in 5 out of 19 cases (26.3%) (Table S1).

### Developmental assessment

**Fig. 1:**
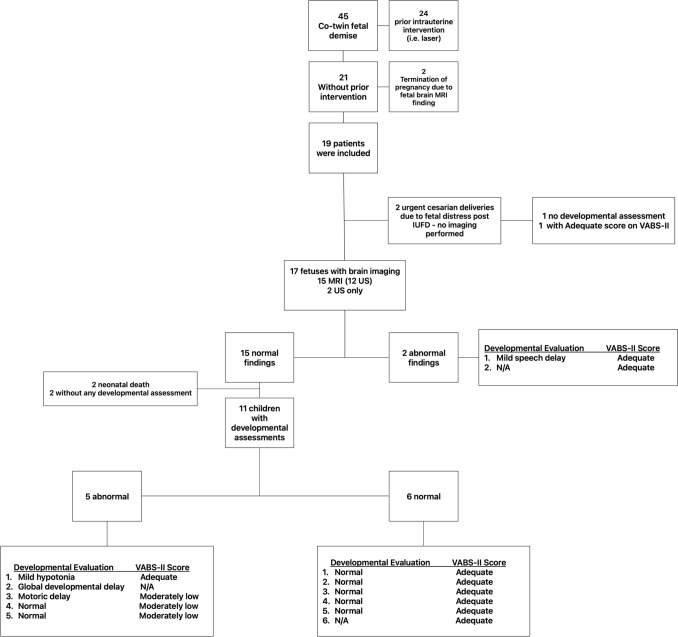
Patient flowchart

14 patients underwent developmental assessment, including 11 who had developmental follow-up at the local *Maternal Child Health Care Clinic*, 13 who completed VABS questionnaires, and 10 who completed both. Two patients did not undergo any developmental assessment. 11 out of 15 fetuses with normal brain imaging underwent developmental assessment. Five out of 11 (45.4%) had an abnormal developmental evaluation, either on clinical developmental assessment, VABS-II, or both. Patient flowchart is depicted in Fig. 1.

### Maternal and child health care clinics assessments

Eleven children underwent developmental assessment that included assessment for personal–social, language, and gross and fine motor skills. Seven had a normal assessment, whereas four patients had abnormal findings, including mild speech delay, mild hypotonia, motor delay, and global developmental delay. Three of these four patients had normal neuroimaging during pregnancy. Ten patients underwent hearing and vision tests, and all were normal.

### Vineland adaptive behavior scales

13 patients agreed to participate and complete VABS-II interview and their scores are detailed in Table 2. The children’s median age at time of completing the questionnaire was 66.0 months (interquartile range (IQR), 37.0–91.0); range 25–130 months). Ten out of 13 had an adequate adaptive behavior composite, whereas 3 had moderately low scores. These included low results in motor and daily living skills. All these three patients had normal fetal CNS imaging. Distribution of all patients’ scores across adaptive levels corresponding to domains and Adaptive Behavior Composite Standard Score are described in Table 3. The pregnancy characteristics of the five patients with normal imaging that had developmental abnormalities are presented in Table 4.

**Table 2:** VABS-II scores

Parameter	Value
Child’s age at VABS (months)	66.0 (37.0–91.0)
Communication score	100.4 ± 11.0
Daily living score	89.2 ± 7.0
Socialization score	92.1 ± 6.5
Motor skills score	94.6 ± 13.4
Adaptive behavior composite	91.4 ± 6.8

Data given as median (interquartile range) or mean ± SD

**Table 3:** Adaptive levels corresponding to subdomain v-scale scores and domain and adaptive behavior composite standard score, and distribution of all patients

Adaptive level	Domains				Sum of all domains	Brain imaging findings
	Communication skills	Daily living skills	Socialization skills	Motor skills		
Low	0	0	0	1	0	
Moderately low	0	*3*	*2*	*3*	*3*	*Normal (3 patients)*
Adequate	*11*	*10*	*11*	*9*	*10*	*Normal (8 patients)* *Abnormal (2 patients)*^*1*^
Moderately high	*2*	0	0	0	0	
High	0	0	0	0	0	

^1^Two patients with abnormal findings:

1 unilateral acute infarction in the caudate nucleus and enlarged cisterna magna; 2 lateral ventricles asymmetry measuring 7 and 10 mm with irregularity and a signal suggesting Grade I IVH

**Table 4:** Clinical characteristics of patients with normal imaging and developmental abnormalities

Patient	Developmental assessment	Chorionicity	MC complication	GA at delivery
1	VABS-II moderately low	MCDA	Uncomplicated	33.1
2	Mild hypotonia	MCDA	Uncomplicated	36.0
3	mild speech delay	MCDA	Uncomplicated	35.1
4	VABS-II moderately low with motoric delay	MCDA	Uncomplicated	26.0
5	VABS-II moderately low	MCMA	TTTS, donor survived	32.1

## Discussion

We investigated the long-term neurodevelopmental outcomes in surviving monochorionic twins following in utero demise of their co-twin, with a particular focus on the predictive value of fetal brain imaging. Our results show that 45.4% of the surviving twins with normal fetal brain imaging had neurodevelopmental impairment. These patients did not share a clear or consistent pattern in terms of chorionicity, presence of monochorionic complications, or gestational age at delivery. While some were born extremely preterm or had MC-related complications such as TTTS, others were delivered in the late preterm period with otherwise uncomplicated courses (Table 4).

Specifically, out of the 17 patients who underwent brain imaging, 88.2% (n = 15) had normal findings. Eleven out of 15 fetuses with normal brain imaging underwent developmental assessment. Of these, the subsequent developmental assessments showed that 54.6% (6/11) of children had normal development. However, our findings also highlight that even with normal imaging, there remains a non-negligible risk of neurodevelopmental impairment. Among the children with normal brain imaging, 45.4% (5/11) exhibited developmental abnormalities, including motoric delay, mild hypotonia, global delay, and moderately low VABS-II scores. Of note, although normal fetal brain imaging did not rule out neurodevelopmental impairment, the degree of developmental impairment in most of these children was mild to moderate. The fact that normal fetal brain imaging did not rule out neurodevelopmental impairment raises the possibility that, in some cases, the morphologic changes associated with fetal brain damage are too subtle to be detected prenatally. This underscores the need for ongoing developmental surveillance in this population, even when fetal brain imaging does not reveal immediate concerns.

The findings also draw attention to the subset of children with abnormal brain imaging. Out of the two patients with abnormal imaging, one child had mild speech delay, and one child had no neurodevelopmental impairment. This suggests that abnormal fetal brain imaging may be associated with a higher risk of neurodevelopmental delays, highlighting the importance of early and ongoing intervention for these children. Regarding postnatal imaging, in cases of intraventricular hemorrhage (IVH), following co-twin demise or extreme prematurity, the timing and underlying mechanism remain difficult to determine. It is unclear whether the IVH resulted from an in utero ischemic insult that was undetected by fetal neuroimaging, or whether it developed postnatally as a consequence of severe prematurity.

The prevalence of neurodevelopmental impairment in this study is consistent with previous research indicating significant risks for surviving twins following co-twin demise in monochorionic pregnancies. Prior studies have reported neurodevelopmental impairments in approximately 26% of surviving twins, with postnatal cranial imaging abnormalities in 34% of cases [3]. Our study’s findings align with previous reports, with 5 out of a total of 19 children (26.3%) in our cohort experiencing neurodevelopmental impairment. However, only 19% (4 out of 21) had imaging abnormalities. These findings reinforce the understanding that monochorionic twin pregnancies carry substantial risks for the surviving twin following a co-twin’s fetal death.

### Strength and limitations

A major strength of this study is the comprehensive approach to developmental assessment, including both objective evaluations and subjective measures through the VABS-II. This dual approach provided a robust and nuanced understanding of the children’s neurodevelopmental status. Additionally, the use of advanced fetal imaging techniques, including diffusion-weighted imaging (DWI) and MRI, enhances the reliability of detecting potential brain abnormalities. Another notable strength is the long-term follow-up of patients, which allowed for the evaluation of neurodevelopmental outcomes beyond the immediate postnatal period and provided valuable insight into the persistence of impairment over time. However, the study has several limitations. The small sample size limits the generalizability of the findings, and the retrospective design may be subject to selection bias. The variation in the timing of imaging and developmental assessments could also affect the results. Furthermore, two patients did not undergo any developmental assessment, which could potentially skew the outcomes if these cases had unique characteristics or outcomes.

Future research should aim to include larger cohorts to validate these findings and enhance their generalizability. Prospective studies with standardized timing for imaging and developmental assessments could provide more consistent and reliable data.

## Conclusion

While normal fetal brain imaging post-co-twin demise in monochorionic pregnancies is generally associated with favorable neurodevelopmental outcomes, a significant risk of neurodevelopmental delay still remains. Recognizing these risks facilitates better clinical consultation, aiding parents in making informed decisions regarding pregnancy management. If the pregnancy is continued, vigilant pre- and post-natal monitoring is essential.

## Supplementary Information

Below is the link to the electronic supplementary material.

Supplementary file1 (DOCX 18 KB)

## Data Availability

No datasets were generated or analyzed during the current study.
