# Supplementary material for: Does normal fetal brain imaging in monochorionic twins following co-twin fetal demise eliminate the risk of adverse neurodevelopmental outcome?
Source: Arch Gynecol Obstet. 2025 Aug 22;312(5):1591–7. doi: 10.1007/s00404-025-08149-6 (PMC12589381; doi:10.1007/s00404-025-08149-6)
Supplement: Supplementary file 1 — Supplementary file1 (DOCX 18 KB) [file 404_2025_8149_MOESM1_ESM.docx]

| **Table S1. Postnatal Head Ultrasound and related** | | | | | |
| --- | --- | --- | --- | --- | --- |
| **Patient** | **Postnatal Head Ultrasound** | **Prenatal Neuroimaging** | **GA at Birth** | **Developmental Assessment** | **VABS-II**  **Score** |
| 1 | normal | normal | 30.5 | normal | Adequate |
| 2 | normal | normal | 29.3 | normal | Adequate |
| 3 | normal | normal | 26.0 | normal | Moderately Low |
| 4 | normal | normal | 34.5 | N/A | Adequate |
| 5 | normal | normal | 34.5 | normal | Adequate |
| 6 | normal | normal | 36.0 | Mild hypotonia | Adequate |
| 7 | normal | unilateral acute infarction in the caudate nucleus and enlarged cisterna magna | 35.1 | mild speech delay | Adequate |
| 8 | normal | normal | 26.0 | normal | Moderately Low |
| 9 | normal | normal | 32.1 | normal | Adequate |
| 10 | normal | not performed | 27.4 | N/A | Adequate |
| 11 | normal | normal | 31.5 | N/A | N/A |
| 12 | normal | normal | 34.0 | N/A | N/A |
| 13 | normal | not performed | 36.0 | N/A | N/A |
| 14 | Cystic PVL | Normal | 26.5 | motoric delay | Moderately Low |
| 15 | Grade I IVH | Suspected Grade I IVH^1^ | 33.1 | N/A | Adequate |
| 16 | Normal (LSV) | normal | 33.2 | normal development | Adequate |
| 17 | Grade III IVH | normal | 29.0 | Global developmental delay | N/A |
| 18 | Grade IV IVH | normal | 25.2 | passed away | |
| 19 | Grade II IVH | normal | 26.2 | passed away | |
| GA – Gestational Age; PVL - Periventricular leukomalacia; LSV- Lenticulostriate vasculopathy; IVH- Intraventricular hemorrhage, VABSII - Vineland-II Adaptive Behavior Scales  ^1^Lateral ventricles asymmetry measuring 7 and 10 mm with irregularity and a signal suggesting Grade I IVH | | | | | |
